# Supplementary material for: Effects of Elicitation on Abeliophyllum distichum Leaf Callus and Changes in Verbascoside Content
Source: Plants (Basel). 2025 May 4;14(9):1386. doi: 10.3390/plants14091386 (PMC12073754; doi:10.3390/plants14091386)
Supplement: Supplementary file 1 [file plants-14-01386-s001.zip › plants-3597539-supplementary.pdf]

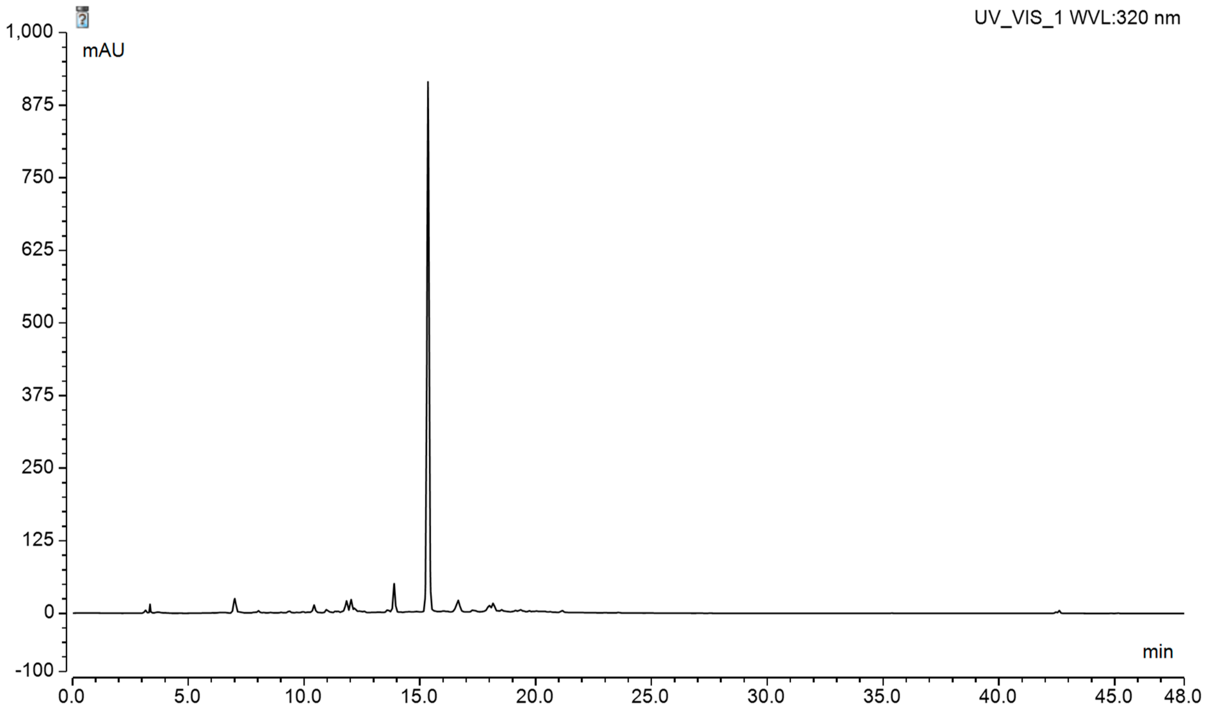

Figure S1. HPLC chromatogram of the control callus cultured in a Petri dish for 4 weeks. The verbascoside peak appeared at a retention time of 15.35 min with an area of 98.78 mAU*min.

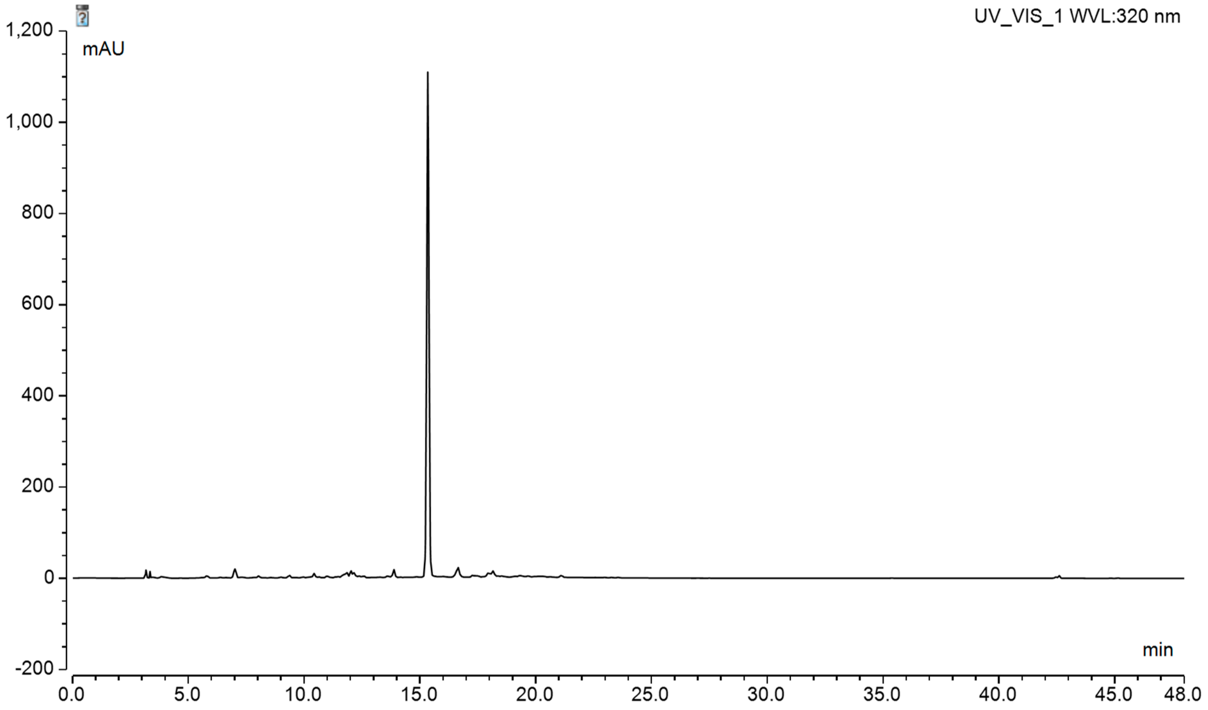

Figure S2. HPLC chromatogram of the callus treated with 50 μM salicylic acid (SA) and cultured in a Petri dish for 4 weeks. The verbascoside peak appeared at a retention time of 15.35 min with an area of 122.62 mAU*min.

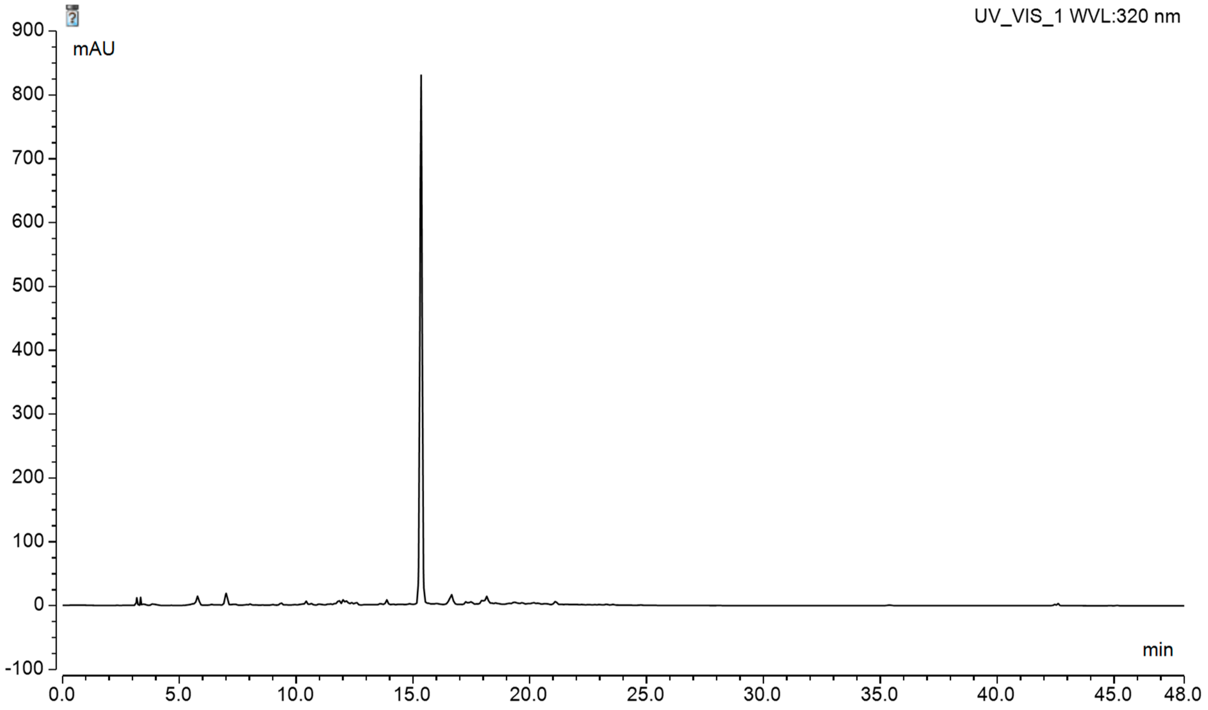

Figure S3. HPLC chromatogram of the callus treated with 100 μM salicylic acid (SA) and cultured in a Petri dish for 4 weeks. The verbascoside peak appeared at a retention time of 15.35 min with an area of 91.65 mAU*min.

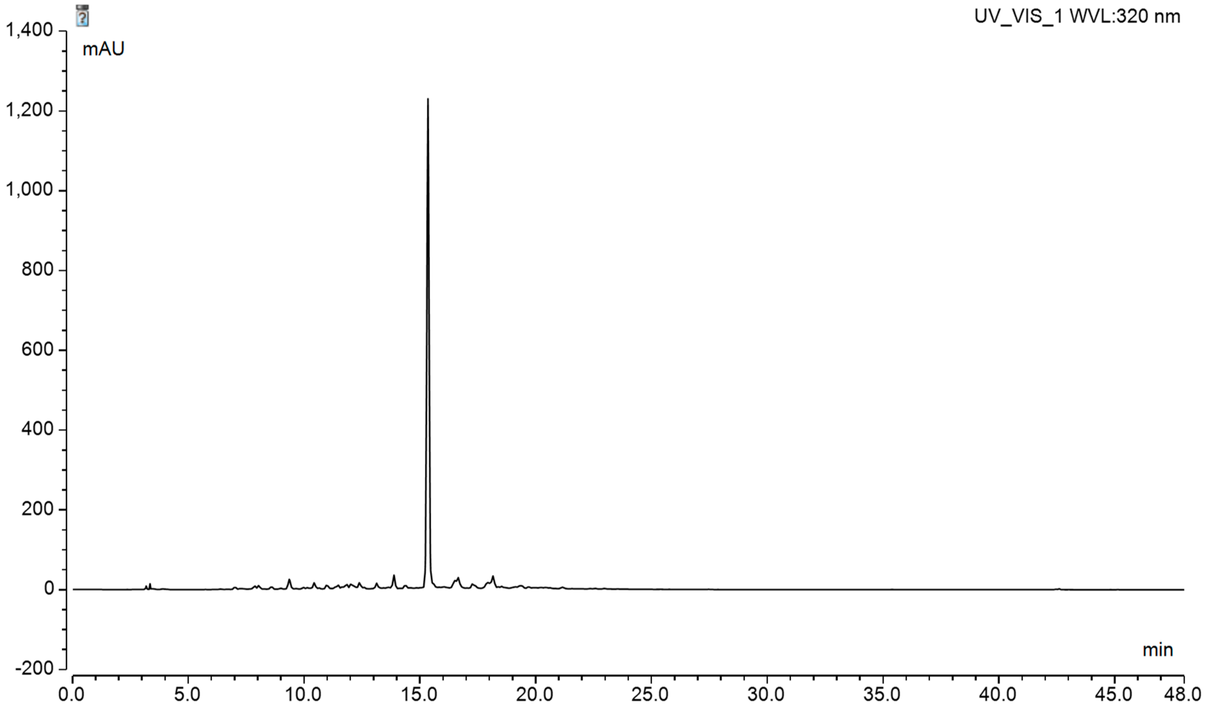

Figure S4. HPLC chromatogram of the callus treated with 50 μM methyl jasmonate (MeJA) and cultured in a Petri dish for 4 weeks. The verbascoside peak appeared at a retention time of 15.35 min with an area of 132.44 mAU*min.

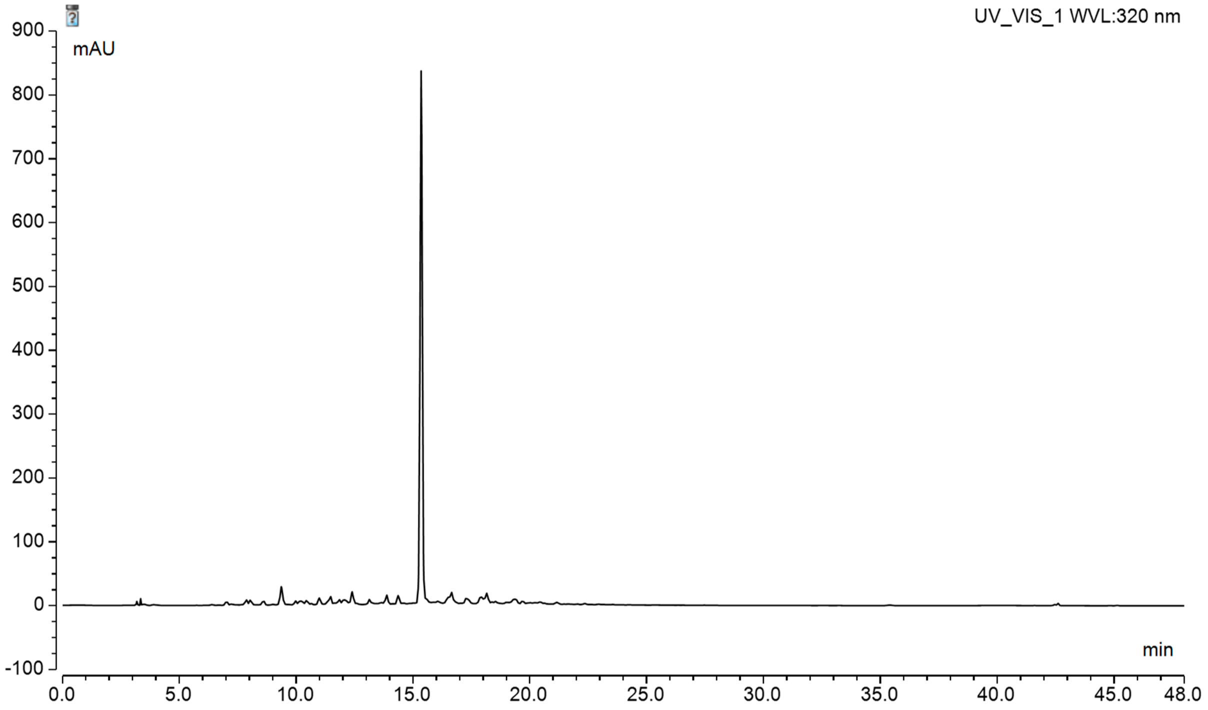

Figure S5. HPLC chromatogram of the callus treated with 100 μM methyl jasmonate (MeJA) and cultured in a Petri dish for 4 weeks. The verbascoside peak appeared at a retention time of 15.35 min with an area of 93.40 mAU*min.

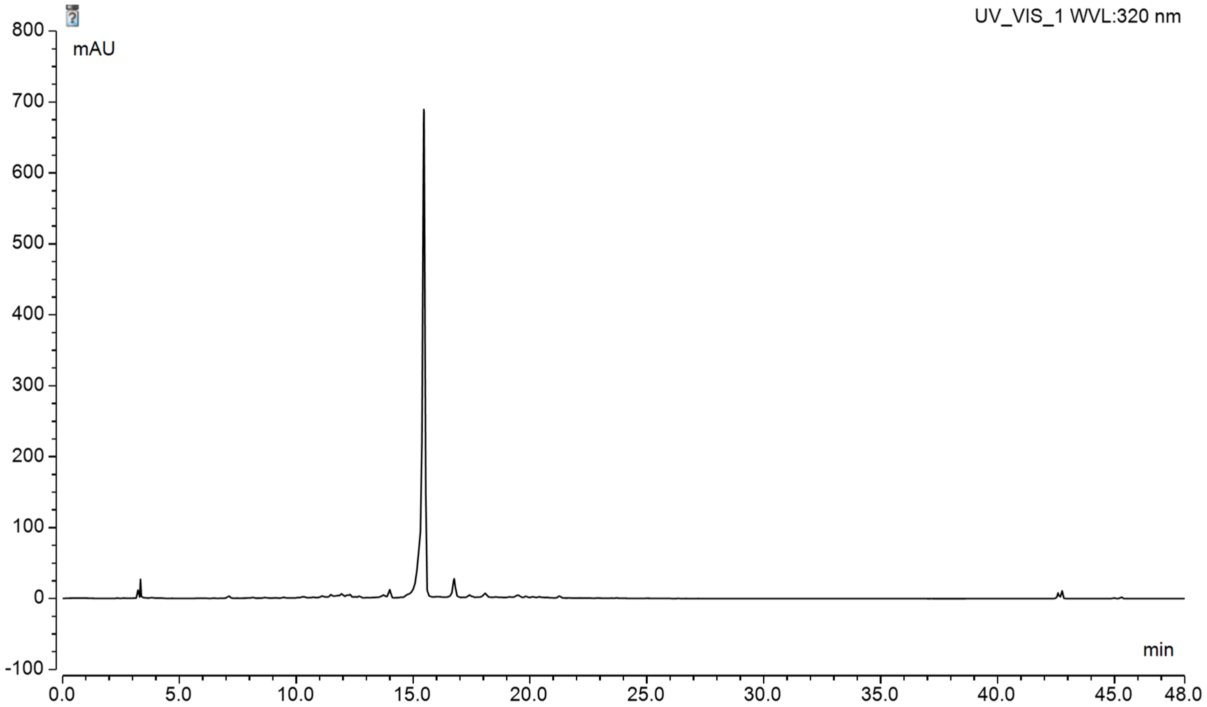

Figure S6. HPLC chromatogram of the control callus cultured in a bioreactor for 4 weeks. The verbascoside peak appeared at a retention time of 15.35 min with an area of 102.82 mAU*min.

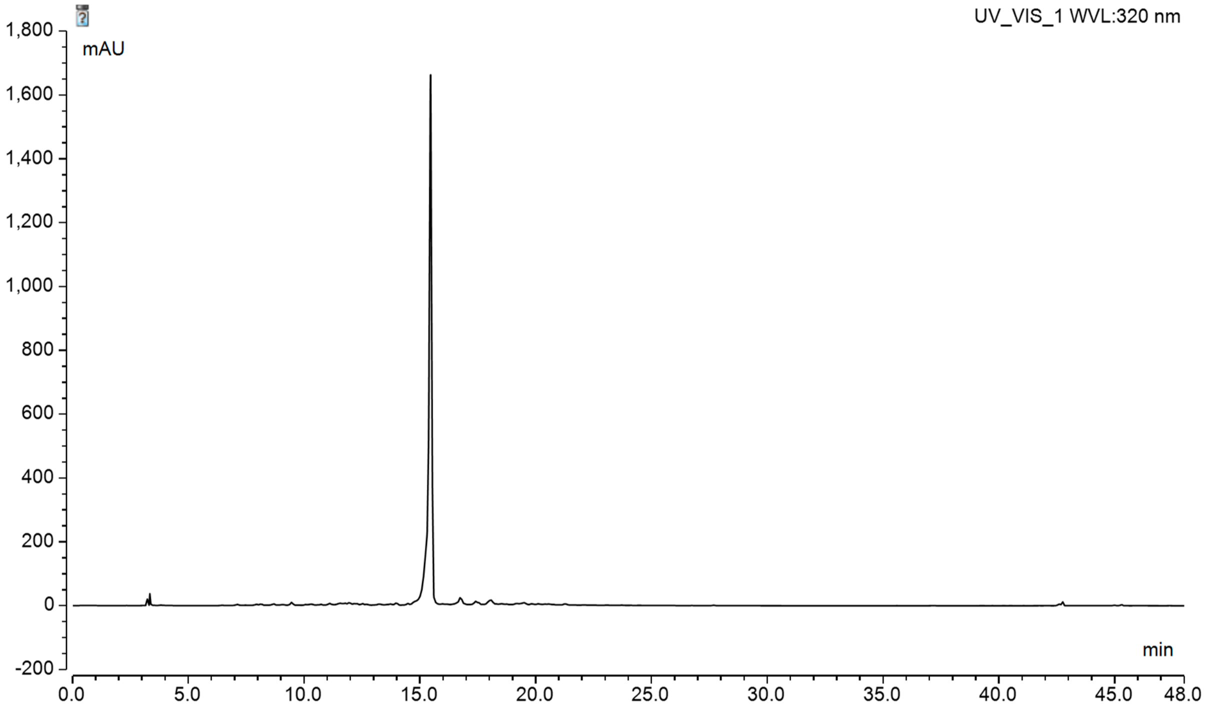

Figure S7. HPLC chromatogram of the callus cultured in a bioreactor for 4 weeks, with 50 μM methyl jasmonate (MeJA) applied for 1 week after 3 weeks of cultivation. The verbascoside peak appeared at a retention time of 15.35 min with an area of 246.53 mAU*min.

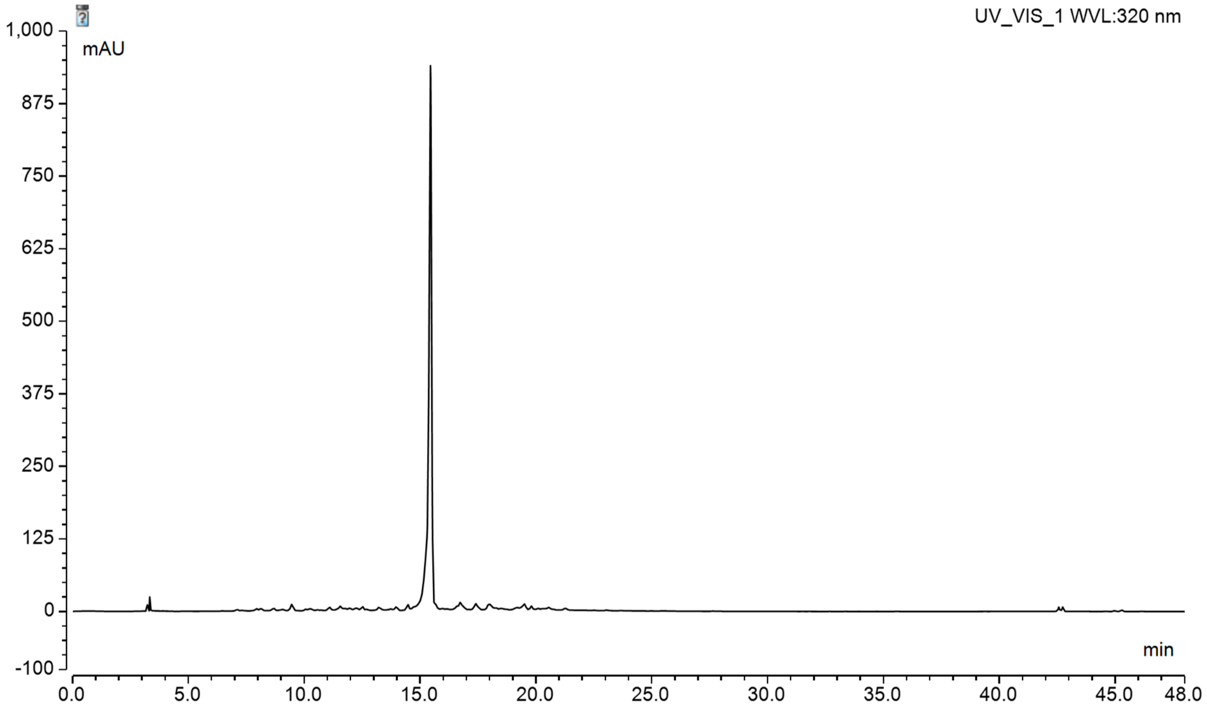

Figure S8. HPLC chromatogram of the callus cultured in a bioreactor for 4 weeks, with 50 μM methyl jasmonate (MeJA) applied for 2 week after 2 weeks of cultivation. The verbascoside peak appeared at a retention time of 15.35 min with an area of 137.41 mAU*min.

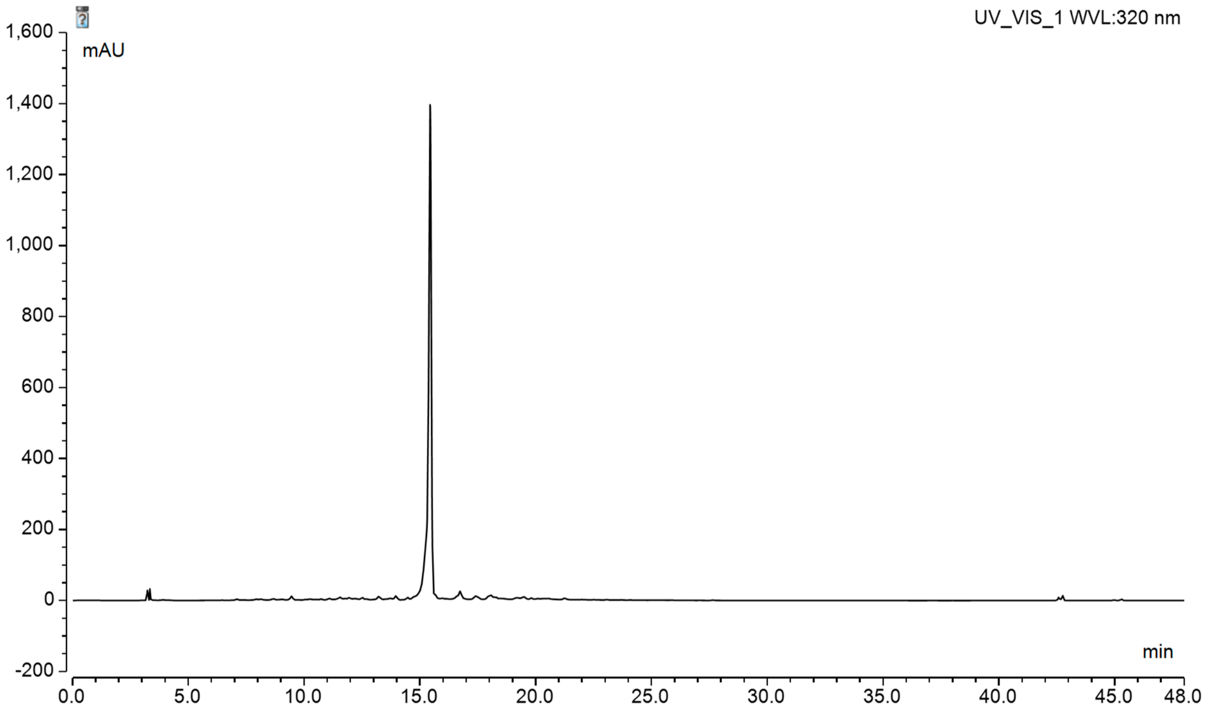

Figure S9. HPLC chromatogram of the callus cultured in a bioreactor for 4 weeks, with 50 μM methyl jasmonate (MeJA) applied for 3 week after 1 weeks of cultivation. The verbascoside peak appeared at a retention time of 15.35 min with an area of 204.46 mAU*min.

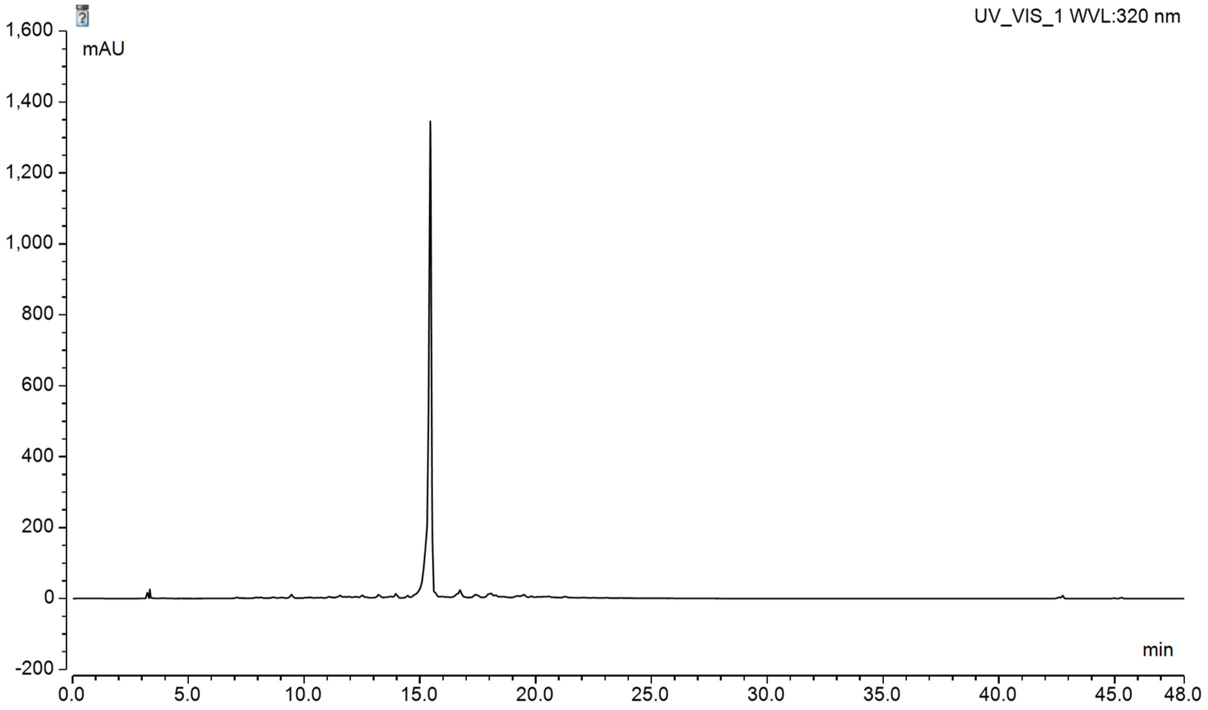

Figure S10. HPLC chromatogram of the callus treated with 50 μM methyl jasmonate (MeJA) for 4 weeks and cultured in a bioreactor. The verbascoside peak appeared at a retention time of 15.35 min with an area of 195.80 mAU*min..
